# Aloe Vera for Tissue Engineering Applications

**DOI:** 10.3390/jfb8010006

**Published:** 2017-02-14

**Authors:** Shekh Rahman, Princeton Carter, Narayan Bhattarai

**Affiliations:** Department of Chemical, Biological and Bioengineering, North Carolina A&T State University, Greensboro, NC 27411, USA; srahman@aggies.ncat.edu (S.R.); plcarter@aggies.ncat.edu (P.C.)

**Keywords:** biomaterials, aloe vera, tissue engineering, regenerative medicine, nanofibers

## Abstract

Aloe vera, also referred as *Aloe barbadensis* Miller, is a succulent plant widely used for biomedical, pharmaceutical and cosmetic applications. Aloe vera has been used for thousands of years. However, recent significant advances have been made in the development of aloe vera for tissue engineering applications. Aloe vera has received considerable attention in tissue engineering due to its biodegradability, biocompatibility, and low toxicity properties. Aloe vera has been reported to have many biologically active components. The bioactive components of aloe vera have effective antibacterial, anti-inflammatory, antioxidant, and immune-modulatory effects that promote both tissue regeneration and growth. The aloe vera plant, its bioactive components, extraction and processing, and tissue engineering prospects are reviewed in this article. The use of aloe vera as tissue engineering scaffolds, gels, and films is discussed, with a special focus on electrospun nanofibers.

## 1. Introduction

Aloe vera is one of the oldest medicinal plants on record due to its biological properties and health benefits. The name aloe vera derives from the Arabic word “alloeh” meaning “shining bitter substance”, while “vera” in Latin means “true” [1]. History has shown us that ancient Chinese and Egyptians used aloe vera to treat burns, wounds, and reduce fever [2]. The aloe vera plant is one of the most studied herbs in the natural products category. Aloe vera is well known for its natural healing capability of the skin and other soft issues. Aloe vera contains over 75 biologically active and naturally-occurring compounds, including polysaccharides, vitamins, enzymes, amino acids, and minerals that work in association with other compounds of the human body to deliver numerous health benefits [3,4].

Aloe vera has been considered a natural healer ever since it was discovered thousands of years ago. However, the use of aloe vera was mainly in the cosmetology and medication. In recent years, there has been increasing interest in natural materials that could be used in tissue engineering. Due to the numerous beneficial effects, the use of aloe vera in tissue engineering has gained importance. The bioactive components in aloe vera have been reported to have antifungal [5], antiseptic [1], antiviral [6], antibacterial [7], anti-inflammatory [8], antioxidant [9], and wound healing [10] properties. Consequently, aloe vera has become an attractive candidate in the field of biomaterials. Aloe vera is especially attractive as a tissue engineering material because aloe vera promotes cell migration, proliferation, and growth [11,12,13,14,15,16,17].

In this review, we highlight the fundamentals of the aloe vera plant and its bioactive components for regenerative medicine and tissue engineering. We present common methods of fabricating aloe vera based scaffolds, gels, and films. Finally, we discuss the influence of aloe vera combined with other biomolecules such as polycaprolactone (PCL), collagen, chitosan, and poly(vinyl alcohol) (PVA).

## 2. Natural Polymer-Derived Biomaterials

Naturally-derived biomaterials are often presumed to have enhanced compatibility with human tissues, the ability to exhibit bioactivity, and the ability to undergo biodegradation [18]. Because of several advantages, natural biomaterials are usually used to replace or restore structure and function of damaged tissues or organs. Natural biomaterials have the ability to adequately support cell attachment, migration, proliferation, and differentiation [19,20]. However, the use of natural materials often has the problem of provoking immune reaction of the host tissues [21,22]. There is also a limitation in the performance of several natural materials in comparison to synthetic materials. Synthetic materials—e.g., synthetic polymers—can be processed easily into a wide range of shapes, whereas natural materials—e.g., natural polymers—cannot be processed easily to obtain desired shapes [23]. In general, mechanical properties and thermal stability of synthetic materials are also better than natural materials [24,25]. Therefore, many of the natural polymers have to be chemically or physically modified before they are used as biomaterials. Blends of synthetic and natural polymers can form a new class of materials with improved mechanical properties and biocompatibility compared with those of single components [23,26,27,28]. The aloe vera plant is a good source of natural polymers that remains to be explored extensively with other biomaterials for tissue engineering applications. Polymer extracts of aloe vera have potential to be used as biomaterials in tissue engineering due to numerous advantages such as biodegradability, oxygen permeability, antioxidant action, and cell proliferation and regeneration. These extracts are also cost-effective and have low toxicity effects.

## 3. Aloe Vera Plant and Its Bioactive Components

**Figure 1:**
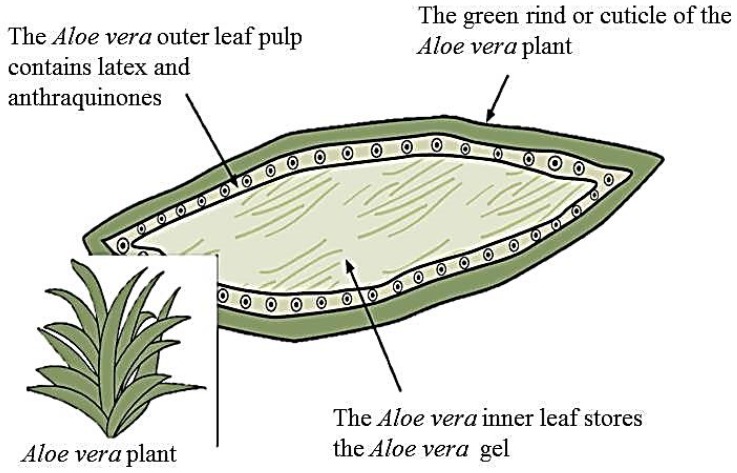
Drawing illustrating the three layers of the aloe leaf.

Aloe vera or *Aloe barbadensis* Miller (family: Liliaceae) is a plant whose components have been thought to have medicinal properties for thousands of years [29]. The plant is considered a succulent species due to its thick leaves that help it to retain water in hot, arid climate [30]. When referring to aloe vera, there is a distinction between each layer of the leaf. Figure 1 shows an image of the plant along with a drawing illustrating the three layers. The inner clear gel, also known as the mucilaginous layer is thought to be responsible for the majority of the plant’s therapeutic properties. Nearly 99% of this layer is water and the rest is made of glucomannans, amino acids, lipids, sterols, and vitamins. On dry basis, aloe gel contains approximately 55% polysaccharides, 17% sugars, 16% minerals, 7% proteins, 4% lipids, and 1% phenolic compounds [2]. The middle layer is comprised of latex, a bitter yellow sap, which contains anthraquinones and glycosides. Molecules from this layer give aloe its laxative effects. Finally, the outer thick layer, or rind, comprised of 15–20 cells, has a protective function and synthesizes carbohydrates and proteins [1]. Vascular bundles inside the rind are composed of xylem and phloem [31]. Xylem helps to transport water and minerals from roots to leaves and phloem helps to transport starch and other small organic molecules. The chemical composition of the aloe plant varies, depending on species, climate, and growing conditions [32]. It has been estimated that there are over 75 bioactive compounds contained in the aloe vera leaf that are listed in Table 1. The multiple compounds have given rise to the many purported benefits of the plant.

Aloe has been described as having anti-inflammatory effects, wound healing properties, radiation damage repair benefits, antibacterial, antiviral, antifungal, antidiabetic, and antineoplastic activities, hematopoietic stimulation, and antioxidant effects [29,33]. The polysaccharides are responsible for the majority of the biological activities observed from the use of the aloe vera plant [34]. However, the biological activities of aloe vera result from a synergistic action of a variety of compounds [33]. The polysaccharides consist of linear chains of glucose and mannose molecules. The major polysaccharides include cellulose, hemicellulose, glucomannans, mannose derivative, and acetylated compounds. Acemannan and glucomannan are considered the two main functional components of aloe vera. Acemannan is composed of a long chain of acetylated mannose, i.e., β-(1,4)-linked acetylated mannose with molecular weights ranging from 30 to 40 kDa or greater [35,36]. The anti-inflammatory effect of aloe is a result of mannose-6-phosphate and acemannan [4]. Glucomannan and acemannan were proved to accelerate tissue regeneration, activate macrophages, stimulate the immune system, and have antibacterial and antiviral effects [36]. These aloe vera constituents are thought to reduce inflammation caused by an increase in prostaglandin synthesis and an increase in the infiltration of leukocytes [37,38]. Acemannan also plays an important role in cellular metabolism by regulating the flow of nutrients and wastes. The sugars, however, are less effective against inflammation caused by allergic reactions. Aloe vera contains anthraquinones—e.g., barbaloin, emodin, anthranol. Anthraquinones, phenolic compounds of aloe leaves, have proven to be effective components, which possess strong anti-inflammatory effects [33], antioxidant effects [9], and antibacterial activities [7]. Barbaloin and other polyphenolic compounds act as antioxidants to inhibit free radicals mediated cytotoxicity and lipid peroxidation [39]. The anti-inflammatory effects of the plant are also a result of the inhibition of enzymes involved in inflammation [8].

## 4. Extraction and Processing of Aloe Vera Gel and Powder

The extraction and processing of gel and powder from aloe vera plant have become a big industry worldwide due to applications in the food, medicine, and cosmetic industries. Fresh gel can be harvested directly from the aloe vera leaves and stored for future use. When this gel is processed, a watery clear fluid with a light amber color comes out. The quality of extraction is determined by species, growing circumstances (e.g., climate, amount of water, fertilization), time of harvest and method of extraction. Extraction of aloe vera gel often involves some processing steps—e.g., crushing, grinding and pressing the entire leaf, or filleting to remove the outer leaf and grinding the gel to produce an aloe juice—followed by various steps of filtration and stabilization [2,40]. The processing steps can alter the properties of polysaccharides by affecting their original structure, which may bring some changes in the proposed physiological and pharmaceutical properties of these constituents [41]. It therefore becomes imperative that a simple but efficient processing technique needs to be developed to improve product quality, and to preserve and maintain almost all of the bioactive chemical entities naturally present in the plant [42]. Time, temperature, and sterilization are also important to preserve the bioactive molecules of aloe vera [2]. Aloe vera leaves start to lose biological activity at six hours following harvest at ambient temperature, and most biological activities are completely lost after 24 h [43].

One of the key factors affecting the functional properties of aloe vera is the handling the aloe leaves after harvesting. The fresh, mature, and mold free leaves are refrigerated within 6 h of harvesting, or directly fed to the processing units [2]. Sehgal et al. reported 8 °C as a storage temperature for 6 h time lapse from harvest to processing [44]. The leaves are washed and disinfected to remove dirt and surface bacteria. In the filleting step, the outer thick layer, or green rind is removed to extract gelatinous fillet. The filleting operation must be completed within 36 h of harvesting the leaves [45]. Gel fillets or entire leaves are crushed or ground within 10–20 min to avoid enzymatic decomposition. The final liquid is followed by various steps of filtration, sterilization, and stabilization. A series of filtration steps are sometimes required to obtain clear aloe vera gel. It was reported that a selected quantity of an oxygen scavenging enzyme such as glucose oxidase is added to the gel to remove oxygen and inhibit the growth aerobic bacteria [46]. Sterilization is usually achieved by exposing the aloe liquid to ultraviolet (UV) light. UV irradiation followed by ultra-filtration was found to be an effective way to sterilize the aloe vera gel [46]. Extraction of gel from the leaves is facilitated by adding cellulose dissolving compounds (e.g., cellulase) and aloe liquid is frequently treated with activated carbon for decolorization and removing aloin and anthraquinones that have laxative effects [47]. The method of centrifugation is found to be effective in the aloe vera gel extraction process [48]. Centrifugal action on aloe gel breaks the chain of sugar molecules surrounded by gel molecules that leads to more gel recovery and fibreless gel [48].

The stabilization of aloe vera gel is usually obtained by concentrating the gel to reduce water content, or drying the gel to make solid powder. The stabilization also can be achieved by adding preservatives and other additives, e.g., sodium benzoate and citric acid [49]. The health benefits of aloe vera are attributed to its biological activities. The aloe vera gel can be concentrated under vacuum without loss of biological activities. The concentration operation must be conducted under 125 mm mercury vacuum at temperatures below 50 °C, and must not exceed two minutes [43]. Spray drying and freeze drying are the most common methods of producing aloe vera powder from gel. Aloe vera powder is less bulky, easier to handle, and less susceptible to spoilage in long term storage, but it is important to ensure the biological activities of the product [40]. The aloe vera gel is generally freeze dried at a temperature between −45 and 30 °C [43]. Spray drying is often carried out at a high temperature (>98 °C) to reduce or eliminate non-desired aloin content [50]. Recently, Medina-Torres et al. [51] and Cervantes-Martinez et al. [52] have investigated the optimum spray drying conditions by using a parallel-flow spray-dryer equipped with a rotary atomizer. The results revealed that inlet flow of 1.5 L/h, temperature of 150 °C, and atomization rate of 27,500 rpm are the spray drying conditions to obtain aloe vera powder with optimum properties. The aloe vera gel or powder must be stored in a dark colored container (preferably glass) to avoid the effect of light on the sensitive bioactive agents.

## 5. Tissue Engineering Prospects of Aloe Vera

Aloe vera has been found to produce beneficial effects on skin wounds, burns, diabetes, obesity, and blood lipids [53]. Today, aloe vera gel is commercially available as an additive to food and drink products [2], and as a moisturizer and healing agent in cosmetics and drugs [1]. The processing of aloe vera gel—derived from the leaf pulp of the plant, for medicinal and cosmetic use—has become a big industry in the United States, one of the largest based on botanicals [54]. There is a great interest in the use of aloe vera for the treatment of damaged tissues, due to its therapeutic properties like anti-inflammatory, antibacterial, antiseptic, and its ability to promote tissue regeneration. Various researchers have conducted experiments to prove the efficacy of aloe vera in regenerative medicine and tissue engineering [55,56,57,58,59,60].

Tissue regeneration is a fundamental response to tissue injury, and this process is achieved mainly by the formation of connective tissue matrix. Collagen is the major protein in the extracellular matrix (ECM) of various connective tissues. It was observed that aloe vera increases the collagen content of the granulation tissue as well as its degree of crosslinking [13]. Aloe vera stimulates fibroblasts for regeneration in a synovial model [17], and enhances tensile strength and collagen turnover in damaged tissues [61]. In another trial using topical application, aloe vera gel stimulated fibroblast activity and collagen proliferation [16]. Glucomannan, a mannose-rich polysaccharide, and gibberellin, a growth hormone, interact with growth factor receptor on the fibroblast, thereby stimulating its activity and proliferation [1]. Hyaluronic acid is a key component of ECM structure and has an important role in the wound healing process. Dermatan sulfate is a proteoglycan of wounds and its release after injury promotes fibroblast growth factor-2 function. It has been reported that aloe vera gel increases levels of hyaluronic acid and dermatan sulfate in granulation tissue of a healing wound [62].

For tissue regeneration, angiogenesis is essentially required to provide oxygen and metabolites to the tissues. Aloe vera can infiltrate into the tissues and increase the transport and activities of biological factors involved in tissue regeneration such as nutrients, cells, enzymes, blood circulation, and oxygen content [58,63]. Different mechanisms are involved in the wound healing effects of aloe vera, including keeping the wound moist, increase of epithelial cell migration, more rapid maturation of collagen, and reduction in inflammation [4,14]. The tissue regenerating function of aloe vera is essentially a result of the synergistic mode of action of many bioactive compounds [64]. Synergy in this case is the interaction of active substances at the biochemical level, as well as the synergistic response of the body to these substances as components of aloe vera consumed. Evidence shows that emodin, one of the derivatives of anthraquinones is also capable of promoting tissue regeneration in a rat model [65]. In an in vitro study, it was found that aloe vera gel increases the proliferation of calf pulmonary artery endothelial cells [66].

Aloe vera plant has been extensively investigated for various purposes in dermatology. The inner gel of aloe vera was shown to inhibit matrix metalloproteinases (MMPs), proteolytic enzymes [67]. These MMPs are released by cellular components of the immune response and are thought to be partially responsible for the tissue destruction observed in chronic periodontitis [68,69]. Aloe, particularly acemannan, has also shown promise in encouraging cells of the periodontium to increase production of their respective ECM [70]. The primary polysaccharide component of aloe vera’s inner gel has specifically been shown to stimulate pulpal fibroblasts, gingival fibroblasts, and cementoblasts [71,72]. A number of glycoproteins present in aloe vera have been reported to increase proliferation of normal human dermal cells [36]. The stimulatory effect on dental cells could potentially lead to improved clinical outcomes of therapies for periodontal diseases.

In addition to favorable interactions with dental cells, aloe vera’s antimicrobial properties make it a favorable candidate for periodontal therapy. Aloe vera has been shown to have antimicrobial activity towards gram-negative bacteria, which are the primary microbes implicated in the etiology of periodontitis [73]. Aloe has anti-inflammatory properties [1], which could mitigate the damage associated with the inflammatory disease—e.g., periodontitis. Finally, the acemannan sugar in aloe vera was shown to encourage the regeneration of periodontium tissues (i.e., alveolar bone, periodontal ligament, and cementum) [70,71,72]. When compared to the traditional antibiotics of vancomycin, bacitracin, methicillin, novobiocin, and erythromycin, the inner gel of aloe vera is effective in inhibiting both gram-negative and gram-positive bacteria [74]. These results suggest that the mucilaginous gel could be used in treating periodontal disease to effectively control the gram-negative anaerobes that cause the disease.

## 6. Aloe Vera Based Tissue Engineering Scaffolds

Tissue engineering scaffolds are potential alternatives to autografts and allografts for use as medical implants. Bioactive molecules such as therapeutic drugs and growth factors can be encapsulated in scaffold materials during fabrication [75]. Most of the scaffolds mimic the structure and biological function of native ECM both in terms of chemical composition and physical structure [76]. The scaffolds, particularly nanofibrous scaffolds, show improved biological response over the bulk materials in aspects of cellular infiltration and in vivo integration. The topography of such scaffolds has been shown to dictate cellular attachment, migration, proliferation, and differentiation, which are critical steps in tissue engineering [77,78,79]. Aloe vera based scaffolds can be fabricated using a variety of techniques [11,12,15]. Electrospinning, and freeze drying (or, thermal induced phase separation) are two techniques, which have been investigated for production of aloe vera based scaffolds. Molecular self-assembly, and phase separation induced by non-solvent are two potential techniques that could be used to fabricate aloe vera based scaffolds [15,80].

### 6.1. Electrospinning Technique

**Figure 2:**
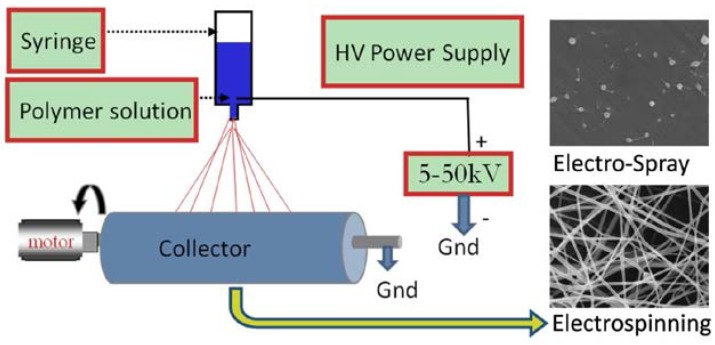
Electrospinning setup adapted with permission from Carter and Bhattarai [89]. Copyright Elsevier, 2013.

Electrospinning is a widely-used technique to produce fibers in the nanoscale. In 1934, Formhals was the first to publish a work concerning the electrospinning process [81]. However, the increased popularity in the use of electrospinning for producing nanofibers for tissue engineering and other applications was sparked by Reneker’s work in the early 1990s [82,83]. During the early 1990s, the technique would also assume its current appellation, becoming a portmanteau of the words ‘electrostatic’ and ‘spinning’ [84]. This technique remains viable primarily because of its simplicity. The electrospinning process involves applying a voltage, typically 1–30 kV, to charge a polymer solution (or melt) loaded into a syringe (Figure 2). This high-applied voltage causes the polymer solution to be sufficiently charged and, the induced charge distributes evenly through the surface. At this point, the solution experiences electrostatic repulsion forces from surface charges and Coloumbic forces from the external electric field [84]. When the electrostatic repulsion forces combined with the Coloumbic force are sufficient to overcome the surface tension of the solution, a stream erupts from the deformed droplet known as a Taylor cone at the end of the nozzle. If polymer cohesion is sufficient, then the stream is elongated and eventually deposited onto a grounded collector. If cohesion or chain entanglement is not sufficient, then electro-spraying or droplet formation usually occurs. Jet elongation happens during the stream’s travel toward the collector. During its flight, the jet undergoes a stretching and whipping process to draw the fiber into an ultrafine, long filament. The solvent simultaneously evaporates, and the fibers are deposited on the grounded collector, thereby creating a non-woven, randomly aligned fibrous mat [84].

The parameters chosen during electrospinning greatly influence the collected fibers. These parameters are typically divided into three categories: polymer parameters, polymer-solution parameters, and parameters of the apparatus. The type of polymer used and its physical properties greatly affect the nanofibers. These properties include the molecular weight, molecular weight distribution, and the branching of the polymer [85]. Solution properties found to have an integral role in fiber formation include viscosity, polymer concentration, conductivity, and surface tension. Important apparatus parameters are applied flow rate, voltage, distance from syringe needle tip to collector, type of collector and whether it is static or dynamic, the type of needle used, and the ambient conditions during electrospinning [82,86,87,88].

Among various techniques, electrospinning technique is relatively simple, inexpensive, and reliable [90]. Electrospun nanofibers are morphological mimics of fibrous components of the native ECM [91,92,93], which makes nanofibrous scaffolds ideal for three-dimensional (3D) cell cultures and tissue engineering applications [94]. Electrospun nanofibrous scaffolds possess an extremely high surface-to-volume ratio, tunable porosity, and malleability to conform over a wide variety of sizes and shapes, and the scaffold composition can be controlled to achieve desired properties and functionality [95]. Due to these advantages, electrospun nanofibrous scaffolds have been widely investigated in the past several years with different synthetic and natural materials for various regenerative medicine and tissue engineering applications. Recently, several attempts have been made to investigate aloe vera incorporated nanofibrous scaffolds.

**Figure 3:**
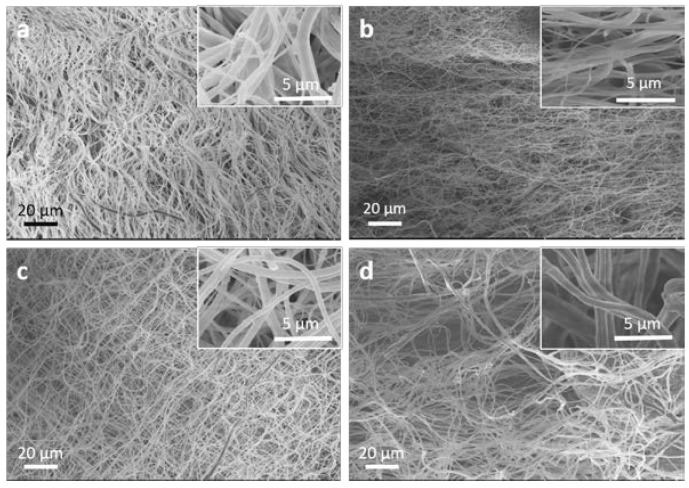
Scanning electron microscope (SEM) images of PCL/aloe vera nanofibers with ratios of (**a**) 100/0; (**b**) 90/10; (**c**) 80/20; and (**d**) 70/30, respectively. The insets show higher magnification images of the structures shown in the corresponding main SEM images. This figure is reproduced with permission from Carter et al. [97]. Copyright Taylor & Francis, 2016.

**Figure 4:**
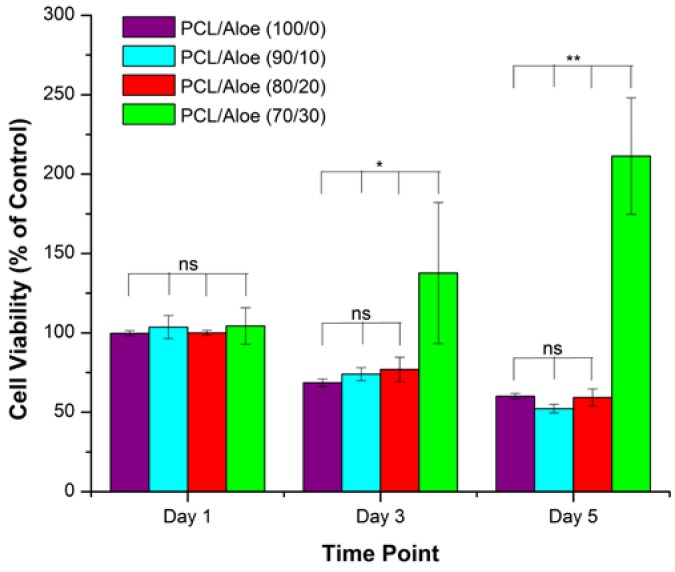
Cell viability results of 3T3 cells grown on PCL/aloe fibers. Statistical significance of *p* < 0.05 is indicated by * and *p* < 0.005 by **. Statistical insignificance of *p* > 0.05 is indicated by ns. This figure is adapted from Carter et al. [97]. Copyright Taylor & Francis, 2016.

Synthetic polymers are used for tissue engineering applications due to their favorable structural, mechanical, and biocompatible behavior [80]. Polycaprolactone (PCL) is a biopolymer widely used for tissue engineering applications, but the major limitation of PCL polymer is the poor cell behavior due to its hydrophobic nature [96]. The authors of this review recently developed a facile technique to blend aloe vera with PCL to create nanofibrous membranes by electrospinning [97]. Nanofibrous membranes with varying composition of PCL to aloe vera were fabricated, and several physicochemical and biological properties were analyzed for guided tissue regeneration (GTR). PCL/aloe vera membranes with ratios from 100/00 to 70/30 showed good uniformity in fiber morphology (Figure 3). PCL/aloe vera membranes with 60/40 ratio or lower showed poor morphology—i.e., “beads on fiber” and weak handle-ability. PCL/aloe vera membranes with ratios from 100/00 to 70/30 also showed suitable mechanical properties and structural stability that are required for GTR membrane. The combination of synthetic PCL with natural aloe vera at a 70/30 ratio significantly improved the cellular compatibility of 3T3 cells on nanofiber membranes (Figure 4), which substantiates that the incorporation of aloe vera can provide adequate support for 3T3 cell growth and proliferation.

Mary and Dev [12] studied the degradation and wettability behavior of aloe vera incorporated PCL electrospun matrices. The aloe vera incorporated PCL matrices degraded at a faster rate compared to PCL matrices, and the hydrophilicity of the fiber mats increased on blending the aloe vera with PCL polymer. Fibroblasts cells cultured on the PCL/aloe vera mats showed rapid proliferation compared to that of pristine PCL mats. In another study, Suganya et al. [15] blended aloe vera with PCL to fabricate electrospun fiber mats for dermal substitutes. The PCL nanofibrous scaffolds with 10% aloe vera showed finer fiber morphology with improved hydrophilic properties and higher tensile strength of 6.28 MPa with a Young’s modulus of 16.11 MPa that are desirable properties for skin tissue engineering. The biological responses of nanofibers were investigated in terms of proliferation and cell morphology of mice dermal fibroblasts. PCL nanofibrous matrix with 10% aloe vera favored cell proliferation compared to other scaffolds with 0% and 5% aloe vera. CMFDA (5-chloromethylfluorescein diacetate) is a fluorescent dye that is used to monitor cell movement or location. Suganya et al. used CMFDA dye to investigate the cellular movement of fibroblasts onto fiber mats. It was found that CMFDA dye expression as well as secretion of collagen and F-actin expression were significantly increased in 10% aloe vera blended PCL scaffolds.

Sridhar et al. [98] studied curcumin, and aloe vera/curcumin composite PCL electrospun membranes for in vitro anticancer activity as drug-eluting implants. The membranes were tested against human breast cancer (MCF7) and lung cancer (A459) cell lines. For the both cell lines, 1% aloe vera and 5% curcumin loaded PCL nanofibers exhibited 15% more cytotoxicity in comparison with the commercial drug 1% *cis*-Platin-loaded PCL nanofibers. Abdullah et al. [99] fabricated aloe vera incorporated poly(vinyl alcohol) (PVA) nanofibers by utilizing electrospinning technique, and they compared PVA/aloe vera nanofibers to PVA nanofibers. The results showed homogenous and linear fiber morphology when PVA is mixed with aloe vera, and the average size of fibers was smaller than the PVA fibers. Ibrahim et al. [100] fabricated chitosan/aloe vera composite nanofibers by electrospinning technique. The average diameter of chitosan/aloe vera composite nanofibers was 183 nm with a range of 140–260 nm. Recently, Garcia-Orue et al. [101] have developed electrospun poly(lactic-*co*-glycolic acid) (PLGA) nanofibrous membranes containing recombinant human Epidermal Growth Factor (rhEGF) and aloe vera extract for wound healing applications. Incorporation of both rhEGF and aloe vera into PLGA nanofibrous membranes revealed several beneficial effects. In vitro cell viability assay showed an improvement of fibroblast proliferation, and in vivo study demonstrated significant acceleration of wound closure and reepithelization. Bhaarathy et al. [102] fabricated composite electrospun nanofibers using copolymer poly(l-lactic acid)-*co*-poly(ε-caprolactone) (PLACL), silk fibroin (SF), and aloe vera for cardiac tissue engineering. The results demonstrated that PLACL/SF/aloe vera nanofibers have more desirable properties compared to PLACL and PLACL/SF nanofibers. PLACL/SF/aloe vera nanofibers were found to be porous, uniform, and beadless. These fibers have an elastic modulus of 7 MPa, a contact angle of 51°, and an average fiber diameter of 188 nm. These properties of PLACL/SF/aloe vera are more desirable to act as flexible cell supporting scaffolds compared to PLACL and PLACL/SF for the repair of myocardial infarction. Cardiac cell proliferation was increased significantly in PLACL/SF/aloe vera nanofibers compared to PLACL nanofibers.

### 6.2. Freeze Drying Technique

Freeze drying is commonly used to fabricate 3D scaffolds for tissue engineering. The scaffolds with porous structure can be fabricated through careful control of the freeze drying process [103,104,105]. 3D scaffolds with porous network are very effective for cell growth, cell migration, and transport of nutrients and metabolic waste [106,107,108]. Freeze drying technique involves three major steps: the solution is frozen at a low temperature (−70 to −80 °C); the frozen sample is located in a chamber in which the pressure is lowered (to a few millibars) through a partial vacuum, known as the primary drying process, in which ice in the material is removed by direct sublimation; and most of the unfrozen water in the material is removed by desorption in a secondary drying process [109]. However, freeze drying is a time and energy consuming technique. For example, freeze drying often takes three to four days to remove solvent, and a lot of energy is consumed to keep vacuum and to maintain the low temperature. If the temperature is not controlled low enough, the porous structure of scaffolds can be damaged due to the interfacial tension caused by the evaporation of solvent.

Jithendra et al. [11] prepared aloe vera blended collagen/chitosan composite scaffolds by freeze drying technique, and several physiochemical properties were characterized using various techniques. The biocompatibility and applicability of composite scaffolds were evaluated in vitro using 3T3 fibroblast cells. The composite scaffolds showed porous architecture with gradual change in their morphology, and tensile properties reduced with increasing aloe vera concentration. The blending of aloe vera to collagen/chitosan increased the thermal stability as well as hydrophilicity of the scaffolds. Cell culture studies on the scaffold showed enhanced growth and proliferation of 3T3 fibroblast cells without exhibiting any toxicity.

### 6.3. Molecular Self-Assembly and Phase Separation Techniques

Spontaneous assembly of small biological molecules is driven by various non-covalent or weak covalent interactions, including electrostatic, van der Waals, hydrophobic interactions, ionic, hydrogen, and coordination bonds [109,110]. These interactions combine the individual molecules to form supramolecular structures [111]. This unique characteristic of nature has inspired many researchers to design novel biomaterials by mimicking natural self-assembled processes [112]. Self-assembly has been utilized for fabrication of various nanofibers using natural and synthetic biomaterials [113,114,115,116,117,118,119,120]. For example, self-assembled nanofibers from natural chitin have been prepared for tissue engineering [121]. Self-assembly is a potential technique for fabricating aloe vera based nanofibers due to natural molecular organization and pattern.

The phase separation technique induced by a non-solvent has been utilized to fabricate porous scaffolds. In this technique, a synthetic or natural polymer is dissolved in a solvent, and phase separation is induced by adding a non-solvent to the solution. This technique is promising for fabricating aloe vera based porous scaffolds due to simplicity and ambient processing conditions. However, induction of phase separation using a non-solvent commonly results in scaffolds with a heterogeneous pore structure, which is not suitable for many tissue engineering applications [109].

## 7. Aloe Vera Based Gels and Films

Aloe vera gel has been investigated for potential use in tissue engineering. Aloe vera gel has been known as a natural healing agent for many years. Wound healing is a complex and dynamic process that involves a cascade of cellular reactions to restore cellular structures and tissue layers. Aloe vera gel has been known to accelerate wound healing [122,123,124], and protect and soothe the skin tissues [125]. The glucomannan, a water soluble polysaccharide in aloe vera, affects fibroblast growth factor and stimulates the activity and proliferation of these cells and, finally improves collagen production and secretion [126]. Mucilage of aloe vera also increases the amount of collagen on the wound site with enhanced transversal connections [127]. The anti-inflammatory property and collagen rising production promote the rearrangement of epithelial tissues [126]. The growth factors present in aloe vera block the wound healing suppression of hydrocortisone acetate by masking the wound healing inhibitors such as sterols and certain amino acids [61].

Hydrogel films are attractive materials for biomedical applications with large potential for cell growth and tissue regeneration. The ability to contain water and stability to withstand the in vivo or in vitro conditions make hydrogel films compatible with biological systems [128]. The exploitation of hydrogel films in cell cultures improves cellular microenvironments by simulating in vivo situations, which are important for cell adhesion, cell attachment, and associated intracellular signaling events [129]. The ability to create biomimic and porous hydrogel films has generated opportunities for engineering different tissues such as skin, cartilage, and nerve tissues [130,131,132,133,134]. Recently, several attempts have been made to investigate the aloe vera incorporated hydrogel films for tissue engineering applications [135,136].

Chitosan is a cationic polysaccharide produced by the exhaustive deacetylation of chitin, a structural element in the exoskeleton of crustaceans and insects. The primary amino groups of the chitosan are responsible for several important properties, such as mucoadhesion, in situ gelation, transfection, permeation enhancement, and efflux pump inhibitory properties [137]. Khoshgozaran-Abras et al. [138] investigated the effect of aloe vera gel incorporation at different proportions on chitosan based films. The physicochemical, mechanical, and color properties of chitosan/aloe vera hydrogel films were evaluated. This study suggested that the incorporation of aloe vera gel into film-forming chitosan solution could have a considerable influence on the properties of the obtained chitosan-aloe vera gel blend films. Incorporation of the aloe gel significantly improves the water solubility, and increases the mechanical properties and, affects the color properties as the higher the gel, the darker the films. In another study, blended membrane films composed of chitosan and aloe vera gel were prepared by utilizing solvent casting technique [60]. It was observed that incorporation of aloe vera gel into chitosan solution increases roughness and wettability, and in vitro cell culture studies confirmed by MTS testing show high cell viability.

Alginate is a biodegradable and biocompatible anionic polysaccharide widely used in wound dressing applications. Pereira et al. [139] investigated the influence of aloe vera on water adsorption and in vitro degradation rate of alginate hydrogel films. It was found that the water adsorption is significantly higher for films containing high aloe vera contents (15% and 25%), and aloe vera significantly increases the degradation rate. Therefore, the incorporation of aloe vera into alginate hydrogel films can tailor the water absorption and degradation rate, which are important properties for tissue engineering applications, particularly for wound dressing. Recently Anjum et al. [140] have developed a two-step process to fabricate a composite material containing nano-silver hydrogels of poly(methacrylic acid), aloe vera, and curcumin. The composite material was evaluated using in vitro antimicrobial and in vivo animal studies. The results suggested that the composite material has an effective antimicrobial nature, and adding aloe vera promotes would healing and infection control. Tummalapalli et al. [141] loaded aloe vera and curcumin into oxidized pectin-gelatin matrices to fabricate a composite wound dressing. The composite wound dressing was found to exhibit very rapid wound healing, and aloe vera showed a strong anti-inflammatory effect and prominent scar prevention.

## 8. Conclusions

Tissue engineering has attracted much attention as a therapeutic tool aiming at replacing or repairing damaged tissues or organs. Aloe vera has a promising future for tissue engineering applications because of its unique and appealing physicochemical and biological properties. Aloe vera based gels, films, and nanofibrous scaffolds can be prepared with good control on their composition, function, and morphology. Aloe vera has been shown to be useful for tissue engineering as a wound dressing material, drug-eluding implant, and scaffold material. Aloe vera and aloe vera based biomaterials have attracted many researchers around the world because of attractive features, including efficacy of bioactive molecules, biodegradability, biocompatibility, hydrophilicity, and production of mimic ECM structure. However, some fundamental aspects of aloe vera for tissue engineering remain to be explored and improved through in vitro and in vivo studies. It is imperative that important technologies need to be developed to confirm the translational issues on the role of bioactive molecules of aloe vera and their real impact on tissue engineering.

## Figures and Tables

**Table 1:** Compounds found in aloe vera [1,4,31,33,34].

Type	Compounds
Anthraquinones/anthrones	Aloe-emodin, aloetic-acid, anthranol, aloin A and B (collectively known as barbaloin), isobarbaloin, emodin, ester of cinnamic acid
Carbohydrates	Pure mannan, acetylated mannan, acetylated glucomannan, glucogalactomannan, galactan, pectic substance, arabinogalactan, galactoglucoarabinomannan, galactogalacturan, xylan, cellulose
Enzymes	Alkaline phosphatase, amylase, carboxypeptidase, carboxylase, catalase, cyclooxidase, phosphoenolpyruvate, cyclooxygenase, superoxide dismutase, lipase, oxidase
Inorganic compounds	Calcium, chlorine, phosphorous, chromium, copper, magnesium, iron, manganese, potassium, sodium, zinc
Non-essential and essential amino acids	Alanine, arginine, aspartic acid, glutamic acid, glycine, histidine, hydroxyproline, isoleucine, leucine, lysine, methionine, proline, threonine, tyrosine, valine, phenylalanine
Proteins	Lectins, lectin-like substance
Saccharides	Mannose, glucose, l-rhamnose, aldopentose
Vitamins	B1, B2, B6, C, β-carotene, choline, folic acid, α-tocopherol
Miscellaneous	Arachidonic acid, γ-linolenic acid, potassium sorbate, steroids (campestrol, cholesterol, β-sitosterol), triglicerides, triterpenoid, gibberillin, lignins, salicylic acid, uric acid
